# The Role of Autophagy in iNKT Cell Development

**DOI:** 10.3389/fimmu.2018.02653

**Published:** 2018-11-14

**Authors:** Guan Yang, John P. Driver, Luc Van Kaer

**Affiliations:** ^1^Department of Pathology, Microbiology and Immunology, Vanderbilt University School of Medicine, Nashville, TN, United States; ^2^Department of Animal Sciences, University of Florida, Gainesville, FL, United States

**Keywords:** invariant natural killer T cells, CD1d, autophagy, thymic development, metabolic switch

## Abstract

CD1d-restricted invariant natural killer T (iNKT) cells are innate-like T cells that express an invariant T cell receptor (TCR) α-chain and recognize self and foreign glycolipid antigens. They can rapidly respond to agonist activation and stimulate an extensive array of immune responses. Thymic development and function of iNKT cells are regulated by many different cellular processes, including autophagy, a self-degradation mechanism. In this mini review, we discuss the current understanding of how autophagy regulates iNKT cell development and effector lineage differentiation. Importantly, we propose that iNKT cell development is tightly controlled by metabolic reprogramming.

## Introduction

Invariant natural killer T (iNKT) cells are an innate-like T cell subset found in most mammalian species. Unlike conventional T cells, iNKT cells recognize glycolipid antigens presented by the MHC class I-like, CD1d molecule. iNKT cells express a semi-invariant T cell receptor (TCR) composed of Trav11 (Vα14)-Traj18 (Jα18) and Trbv13-2, Trbv29, or Trbv1 (Vβ8.2, −7, or −2) in mice (1–3) or TRAV10 (Vα24)-TRAJ18 (Jα18) and TRBV25-1 (Vβ11) in humans (1, 4, 5). Analogous invariant TCR chain usage was found in other mammals such as rats (6) and pigs. iNKT cells can be universally activated with the prototypical iNKT cell antigen α-galactosylceramide (α-GalCer) that was originally isolated from the marine sponge *Agelas mauritianus* (7). Once activated, iNKT cells provide a universal source of T cell help primarily through the rapid production of multiple effector cytokines capable of transactivating an array of immune cells (8, 9). In humans and animal models, α-GalCer has been used to therapeutically target iNKT cells to induce multiple profound effects during different pathological conditions, including cancer, autoimmunity, and infectious disease (8, 10–14).

**Figure 1:**
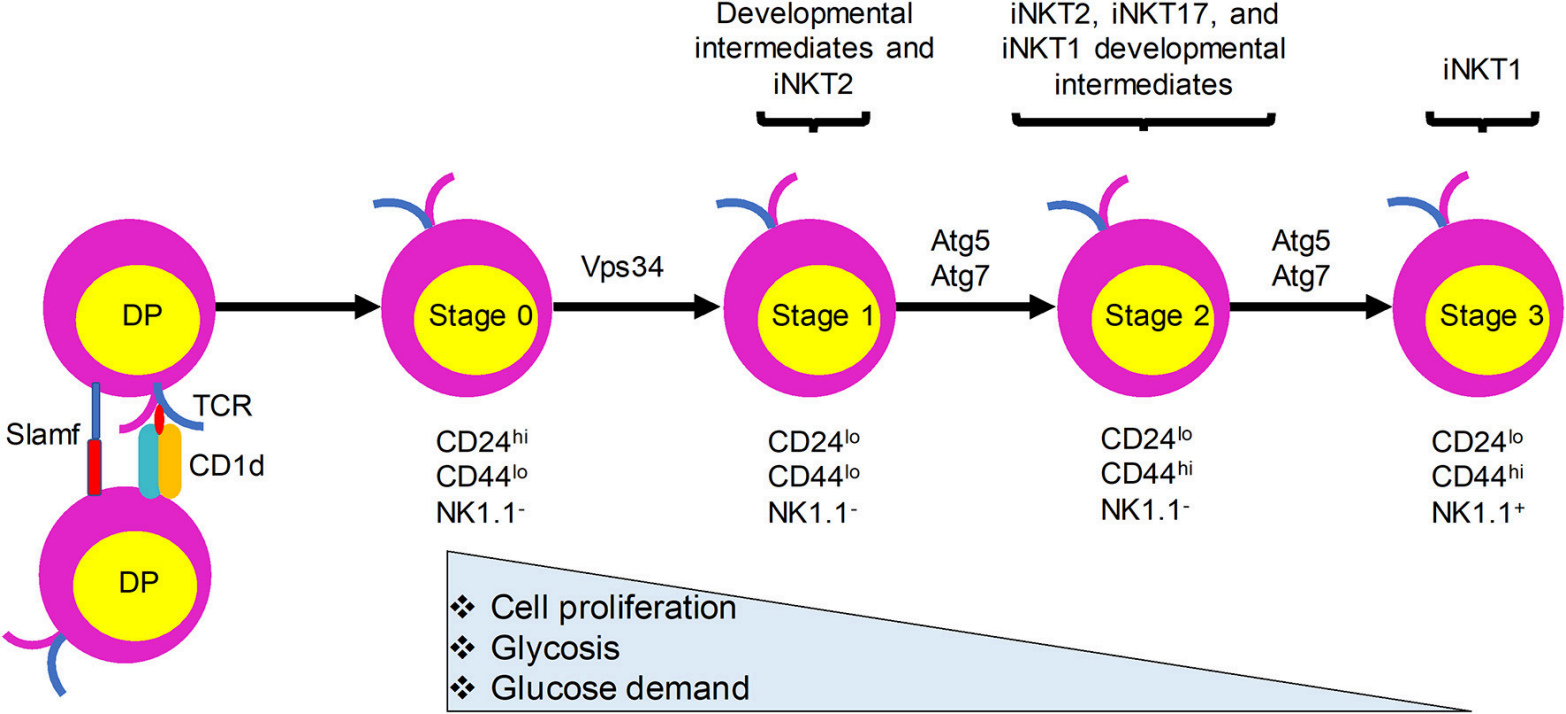
iNKT cells undergo metabolic switching during development and differentiation to meet their changing energy demands. iNKT cells originate from CD4^+^CD8^+^ double positive (DP) thymocytes that express the invariant TCR. They are positively selected by CD1d-expressing DP thymocytes. Immature iNKT cells from DP thymocytes undergo four maturation stages characterized by differential surface expression of CD24, CD44, and NK1.1. Proliferation rate and energy demands decrease as iNKT cells progress from stages 0 and 1 to the more quiescent stages 2 and 3. This transition is accompanied by increased autophagy. Ablation of autophagy genes Atg5, Atg7, or Vps34 in iNKT cells leads to defects in the transition to a quiescent state after population expansion of thymic iNKT cells.

Like the development of conventional T lymphocytes, iNKT cell development depends on somatic DNA recombination and selection in the thymus. CD1d presentation of endogenous ligands is critical for iNKT cell development and animals lacking CD1d have no detectable iNKT cells (15–17). In sharp contrast with conventional T cells, which require MHC expression by thymic epithelial cells for their development, iNKT cells are positively selected by CD1d-expressing CD4^+^CD8^+^ double positive (DP) thymocytes (16, 18) (Figure 1). Nevertheless, a recent study provided evidence that a fraction of iNKT cells develop from late CD4^−^CD8^−^ double negative (DN) stage thymocytes, bypassing the DP stage (19). Negative selection of iNKT cells is not yet clearly defined. Evidence showing that overexpression of CD1d on thymic stromal cells, dendritic cells (DCs), or DP thymocytes in transgenic mice resulted in a variable reduction in the number of iNKT cells suggests that iNKT cells are susceptible to negative selection during their development (20, 21). After the initial selection, iNKT cells transit through four maturation stages, each characterized by sequential acquisition of surface markers: stage 0, CD24^+^CD44^−^NK1.1^−^; stage 1, CD24^−^CD44^−^NK1.1^−^; stage 2, CD24^−^CD44^+^NK1.1^−^; and stage 3, CD24^−^CD44^+^NK1.1^+^ (22, 23). iNKT cells become functionally competent to respond to TCR engagement during their maturation in the thymus. Functionally, thymic iNKT cells can be subdivided into iNKT1, iNKT2, and iNKT17 subsets according to their expression of particular transcription factors, surface markers, and cytokines that are expressed by conventional CD4^+^ T helper (Th) cell subsets (Th1, Th2, and Th17 cells, respectively). Although the relationships between the different stages of iNKT cells and their subsets remain to be fully explored, stage 1 iNKT cells comprise mainly progenitor cells and include cells with the capacity to produce interleukin (IL)-4 that may be related to iNKT2 cells, stage 2 cells likely include all three subsets, and stage 3 cells predominantly include iNKT1 cells (Figure 1). Recent studies have provided evidence that TCR signaling strength governs this iNKT cell subset development, with strong signaling favoring iNKT2 and iNKT17 cell development (24, 25). In addition to these subsets, iNKT follicular helper cells and iNKT10 cells have been identified that resemble T follicular helper cells and regulatory T cells, respectively. Recent studies have revealed a critical role of autophagy, a cellular self-degradation mechanism, in iNKT cell development and function. Here, we review these findings in the context of changes in the metabolic status of developing iNKT cells.

## Signaling pathways that control iNKT cell development

Many signaling proteins and transcription factors are important for iNKT cell development and/or function. Deficiency of the invariant Vα14 TCR or its ligand CD1d results in a failure in iNKT cell generation (7, 17, 26). Runt-related transcription factor 1 is critical for the ontogeny of functional iNKT cells (18). The E protein transcription factor, HEB, is essential for iNKT cells to develop at their earliest developmental stage. This HEB-mediated regulation, in part, is controlled by modulating the expression of retinoic acid receptor-related orphan nuclear receptor gamma t, a possible HEB target, and the anti-apoptotic molecule Bcl-xL (18, 27–29). Once committed to the iNKT cell lineage, multiple other molecules are required for iNKT cell maturation. TCR engagement activates phospholipase Cγ1, which further leads to production of diacylglycerol (DAG) and inositol-1,4,5-trisphosphate (IP3), both of which are critical for iNKT cell development. DAG induces activation of the Ras guanyl nucleotide-releasing protein 1-Ras-extracellular signal-regulated kinase 1/2 pathways and is involved in early iNKT cell development, as well as, late iNKT cell maturation (30). DAG kinases, which are important for controlling intracellular DAG concentration and therefore negatively regulate DAG signaling, are also critical for iNKT cell development (31). IP3 activates the Ca^2+^-calcineurin-NFAT pathway and regulates the generation of stage 1 and 2 iNKT cells via the early growth response protein 2 (Egr2) (32). Egr2 directly binds to the *Zbtb16* promoter and activates transcription factor promyelocytic leukemia zinc finger (PLZF) that supports iNKT cell transition from stage 1 to stage 2 (33, 34). PLZF is thought to control the innate phenotype and is also expressed by MAIT cells, γδ T cells, and innate lymphoid precursor cells in the fetal liver and adult bone marrow (35–37).

Signaling lymphocyte-activation molecule (SLAM) receptors are critical for early iNKT cell maturation (38). Homotypic interactions of SLAM molecules, Slamf1, and Slamf6 and the downstream recruitment of SLAM adaptor protein and the Src kinase Fyn control formation of the stage 0 iNKT cell lineage by activating the NF-κB signaling cascade (39). Other transcription factors including c-Myc, T-bet, Id2, and GATA-3 were shown to regulate different stages of iNKT cell maturation after they migrate to peripheral lymphoid tissues (29). Many additional transcription factors and signaling molecules such as thymocyte selection-associated HMG box protein, Notch signaling, lymphoid enhancer factor, mechanistic target of rapamycin (mTOR), etc., have been reported to impact iNKT cell development and effector function (29, 40, 41). In addition to these pathways, autophagy and autophagy-related pathways also have been reported to be involved in iNKT cell development.

## A brief introduction to autophagy

Autophagy is a highly conserved cellular degradation process. It has been defined as an “auto-digestive” process that promotes the degradation of cytoplasmic proteins and damaged organelles by lysosomes (42). The resulting degradation products are then used in cellular remodeling and in regenerating molecular building blocks during conditions of stress. In this review, we focus on macroautophagy, hereafter referred to as autophagy. The autophagy pathway is tightly regulated by various factors, including nutrient starvation, hypoxia, mitochondrial toxins, and oxidative stress. Over 30 autophagy-related gene (Atg) products that were initially identified in yeast but are largely conserved in higher eukaryotes orchestrate this degradative process. The autophagy process consists of four distinct phases: nucleation, elongation, fusion, and degradation. The morphological hallmark of autophagy is formation of a double-membrane vesicle, termed autophagosome, which is generated in a step-wise manner (42). Nutrient starvation initiates autophagy by inducing dissociation of mTOR from the mTOR substrate complex (ULK1/2, Atg13, FIP200, and Atg101). This dissociation triggers autophagosome nucleation and elongation and leads to recruitment of the class III phosphatidylinositol-3-OH kinase complex, encompassing vacuolar protein sorting (Vps) 34, Vps15, Beclin1, and Atg14, which phosphorylates phosphatidylinositol to generate phosphatidylinositol 3-phosphate (PI3P), a phospholipid critical for membrane trafficking processes. The generation of PI3P leads to recruitment of two ubiquitin-like proteins and initiates the formation of autophagosomes. Briefly, the Atg12 (ubiquitin-like protein)-Atg5-Atg16 complex in conjunction with Atg9 mediates formation of pre-autophagosome structures. During this process, microtubule-associated protein 1A/1B-light chain 3 (LC3, another ubiquitin-like protein) is conjugated to phosphatidylethanolamine with the assistance of Atg4, Atg7 (E1-like enzyme), and Atg3 (E2-like enzyme) to form LC3-II, and associates with newly formed autophagosome membranes until they fuse with lysosomes. The generation of LC3-II is frequently used for monitoring autophagy (43, 44). Upon fusion with lysosomal membranes forming an autolysosome, the autophagic body is degraded by lysosomal esterases, lipases, and proteases and recycled to build new cellular components and energy.

Impaired autophagy is linked to many different diseases, including cancer, inflammatory bowel disease, neurodegeneration, and various cardiovascular, pulmonary, and infectious disorders (45). Autophagy is also implicated in multiple cellular processes, including cell development, survival, and differentiation (46). In the immune system, autophagy plays a critical role in regulating the development of innate and adaptive immunity (47).

## Autophagy in T lymphocyte development

Autophagy is crucial for normal T cell development, activation, and differentiation (48). Briefly, at the precursor stage of T cell development, autophagy regulates hematopoietic stem cell self-renewability and quiescence, which is mediated, at least in part, by the effects of autophagy on reactive oxygen species levels (49). During T cell development in the thymus, autophagy influences thymocyte selection by regulating peptide presentation in stromal cells and professional antigen-presenting cells, which affects survival or proliferation of CD4^−^CD8^−^ DN thymocytes and/or their transition to the DP stage (50). Autophagy is also essential for the maintenance of T cells, especially the long-term survival of naïve T cells in the periphery, via regulation of organelle homeostasis (50). In addition, autophagy is increased in activated T cells (51, 52). Impaired autophagy is associated with T cell malfunction and subset redistribution (50, 53).

## Autophagy influences iNKT cell development and function

### Atg5 and Atg7

Elongation of autophagic vacuoles requires two ubiquitin-like conjugation systems, with critical roles for Atg5 and Atg7. Deficiency in either of these factors blocks most autophagic processes (54, 55). The development and function of iNKT cells were investigated in mouse models with hematopoietic or T cell-specific deletion of Atg5 or Atg7 (56, 57). iNKT cells from these animals displayed an immature phenotype in the thymus (56). Both Atg5- (57) and Atg7-deficiency (56) resulted in defective stage 3 iNKT cell development. These defects correlated with reduced secretion of interferon (IFN)-γ and IL-17, but no change in IL-4 when the thymocytes were cultured with α-GalCer *in vitro* (56) or decreased production of IFN-γ and IL-4 when α-GalCer was injected *in vivo* (57).

Mechanistically, the Atg7-mediated regulation of iNKT cell development was T cell-intrinsic rather than through the presentation of exogenous or self-lipid antigens by thymocytes or bone marrow-derived DCs to iNKT cells (56, 57). Loss of Atg7 also led to reduced expression of Bcl-2, Egr2, and PLZF in iNKT cells. Consistent with these findings, Atg5^−/−^ iNKT cells showed increased cell death coupled with cell cycle arrest and elevated mitochondrial stress (57). Thus, the defect in survival of iNKT cells in these animals might be due to a combination of increased apoptosis (56) and/or autophagy-dependent regulation of cell cycle progression (58).

During development, iNKT cells undergo metabolic switching and require catabolic processes and autophagy for their transition to a quiescent state after cell number expansion from stage 0 to stage 3 (56, 57). More glucose is required for the stage 0 and 1 iNKT cells than for the more mature stage 2 and 3 iNKT cells, which rely more on increased autophagy (56). Overall, these results indicate that Atg5- and Atg7-dependent autophagy is required for iNKT cell development, especially at later stages of the maturation process, with the strongest effects seen on iNKT1 cells, followed by iNKT17 and iNKT2 cells. If and how these effects are related to the differential TCR signaling requirements observed for these distinct subsets remains to be determined.

### Vps34

Vps34 and its binding partner Beclin1 are important for the initiation of autophagy in yeast (59). However, the function of Vps34 in mammalian cells has been controversial, with contributions to autophagy, phagocytosis, endocytosis, and intracellular vesicle trafficking (60–62). We demonstrated that T cells from mice with a T-cell specific deletion of Vps34 showed profound defects in autophagic flux (53). In agreement with the previous findings, Vps34 deletion in T cells significantly impacted T cell homeostasis and function (53). Additionally, Vps34^−/−^ iNKT cells exhibited a developmental blockade at stage 0 (53). Similar to the results in Atg5^−/−^ and Atg7^−/−^ iNKT cells, the restricted iNKT cell development in conditional Vps34^−/−^ mice was T cell-intrinsic and independent of CD1d-restricted antigen presentation (53). The reduced iNKT cell frequency in these animals also correlated with a reciprocal increase in natural killer cells in the spleen, liver, and lymph nodes. Because deletion of Vps34 in T cells, heart, or liver results in the loss of versatile cellular functions besides canonical autophagy (53, 62), it is likely that the role of Vps34 in iNKT cell development is more complex than its influence on autophagy alone. Therefore, the role of Vps34-mediated functions in iNKT cell development may be distinct from those of Atg5 and Atg7. This difference is also illustrated by the finding that deletion of Atg5 or Atg7 genes caused a blockade in stages 2 and 3 iNKT cell development, whereas Vps34-deficiency caused a blockade at stage 0.

### Other autophagy regulators

**Figure 2:**
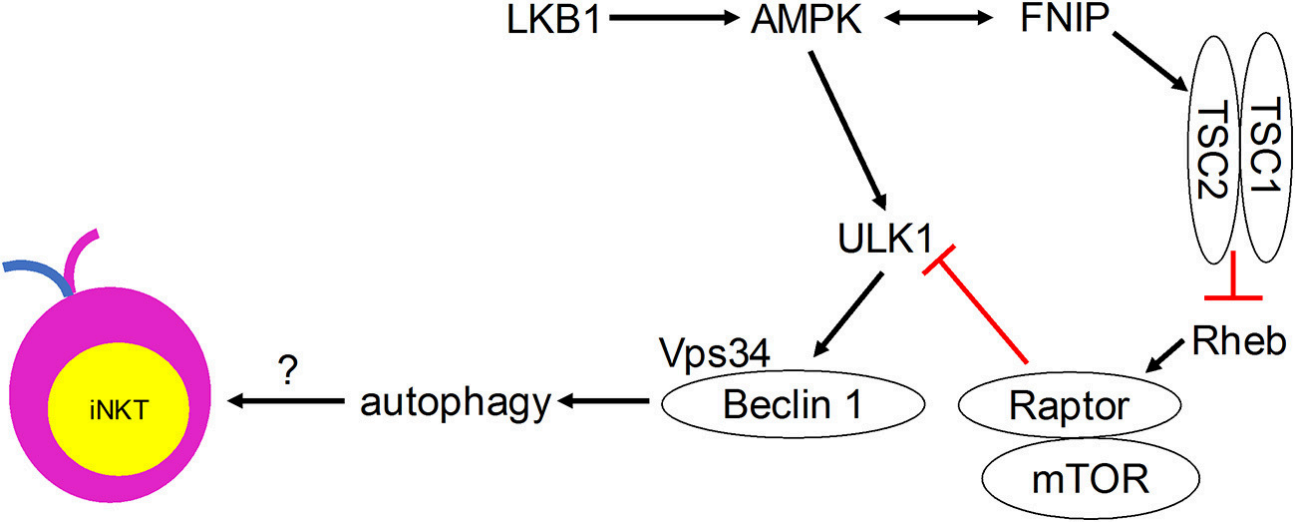
Metabolic signaling pathways that control iNKT cell development in a manner that may involve autophagy.

Although direct evidence is lacking, many autophagy regulators have been shown to play critical roles in mediating iNKT cell development (Figure 2). Ablation of the metabolic homeostasis regulator of early T cell progenitors liver kinase B1 [LKB1, also known as the upstream kinase of AMPK cascade (63)] erased the development of all iNKT cells in the thymus (64). Importantly, CD1d-expressing human B lymphoblastoid cells treated with AMPK agonists were able to induce iNKT cell activation to levels comparable to α-GalCer treatment (65). However, conditional AMPKα deletion in T cells did not affect thymic iNKT cell frequencies, although iNKT cell subsets and function remain to be investigated (64). AMPK-interacting protein Fnip1 is also critical for iNKT cell development to stage 3 by maintaining metabolic homeostasis in response to metabolic stress (66). Similarly, mTOR signaling negative regulator tumor suppressor tuberous sclerosis 1 (Tsc1) is crucial for iNKT cell development (67, 68). Mice lacking Tsc1 showed markedly reduced iNKT cell frequency and absolute cell numbers in spleen and liver (67), and displayed a developmental block of iNKT cell differentiation at stage 2, with decreased IFN-γ- and increased IL-17-secreting iNKT cells (68). This Tsc1-induced regulation resulted from increased mTORC1 activity, as rapamycin treatment partially rescued reverted IL-17-secreting iNKT cell predominance to IFN-γ-secreting iNKT cell predominance in Tsc1-deficient mice (68). In other studies, mTOR was selectively required for thymic iNKT cell development and mTOR-deficiency led to accumulation of stage 0 iNKT cells in the thymus (69), whereas Raptor-deficiency led to severe iNKT cell maturation blockade between thymic stages 1 and 2 (69, 70). The differences in the developmental blockade of iNKT cells between mTOR- and Raptor-deficient animals suggest that mTORC1-independent mTOR pathways such as mTORC2 might be involved in regulating iNKT cell development. These results also suggest that adequate level of mTORC1 is required for iNKT cell development. Taken together, these findings indicate possible regulatory roles for the induction of autophagy downstream of the Tsc-mTOR axis in iNKT cell development.

In addition to metabolic regulators, the role of histone deacetylases (HDACs) in iNKT cell development has been investigated. HDACs are histone-modifying enzymes that mediate removal of acetyl groups from proteins and are strongly involved in the regulation of autophagy [reviewed in (71)]. Deletion of Hdac3 in DP thymocytes completely blocked iNKT cell development without influencing conventional T cell development (72). Loss of Hdac3 in iNKT cells led to a severe reduction of iNKT cells, particularly at stage 3 (73). This depletion of iNKT1 cells was associated with reduced autophagy, although independently of Atg7 and p62 expression, and decreased GLUT1, CD71, and CD98 nutrient receptor expression (73).

TBK-binding protein 1 (Tbkbp1) is a protein with undefined physiological function but physically interacts with the protein kinase TBK1 (74). A recent study identified Tbkbp1 as a crucial regulator of autophagy stimulated by the cytokines IL-15 and IL-2 (75). This Tbkbp1-mediated autophagy regulation was achieved through activation of ULK1 by antagonizing the inhibitory action of mTORC1. Tbkbp1 deficiency caused a T cell-intrinsic selective loss of stage 3 iNKT cells and a relative accumulation of stage 1 and 2 iNKT cells (75). The predominant loss of stage 3 iNKT cells in Tbkbp1-deficient mice was likely contributed by reduced autophagy, as autophagy inhibitor 3-methyladenine caused a high level of apoptosis in both wild-type and Tbkbp1-deficient mice, even in the presence of IL-15. Receptor for activated C kinase 1 (RACK1) is an adaptor involved in multiple intracellular signaling pathways and is important for the assembly of the autophagy-initiation complex (76). RACK1-deficiency blocked thymic iNKT cell development and migration of mature iNKT cells to peripheral lymphoid organs (77).

## Concluding remarks and future outlook

Autophagy, a process that is regulated by the metabolic status of cells, is critically important for iNKT cell development. Quiescent T cells can use autophagy to break down intracellular components to supply molecules for oxidative phosphorylation (78). iNKT cells acquire a memory phenotype while developing in the thymus. They undergo metabolic switching during development and differentiation to meet their changing energy demands, with stage 2 and 3 iNKT cells staying in a more quiescent state than the more proliferative stage 0 and 1 iNKT cells. We propose that autophagy is required for this metabolic switch (Figure 1). Immature proliferative iNKT cells exhibit high glucose uptake, and this high demand for glucose is reduced upon maturation, which is accompanied by diminished proliferation and increased autophagy. Not surprisingly, ablation of autophagy genes Atg5, Atg7, or Vps34 in iNKT cells led to a defective transition to a quiescent state after population expansion of thymic iNKT cells. However, how autophagy might be regulated by upstream metabolic regulators during iNKT cell development remains to be determined (Figure 2). More research is required to elucidate the role of other autophagy-related gene products in iNKT cell development and maturation. Whether autophagy can control the development and differentiation of other innate-like T cells marked by PLZF expression also remains an open question.

## Author contributions

GY wrote the first draft, and GY, JD, and LVK edited the manuscript.

### Conflict of interest statement

The authors declare that the research was conducted in the absence of any commercial or financial relationships that could be construed as a potential conflict of interest.

## Abbreviations

α-GalCer: α-galactosylceramide
Atg: autophagy-related gene
DAG: diacylglycerol
DC: dendritic cell
DN: double negative
DP: double positive
Egr2: early growth response protein 2
HDACs: histone deacetylases
IFN-γ: interferon-gamma
IL: interleukin
iNKT: invariant natural killer T
IP3: inositol-1,4,5-trisphosphate
LC3: microtubule-associated protein 1A/1B-light chain 3
LKB1: liver kinase B1
mTOR: mechanistic target of rapamycin
PI3P: phosphatidylinositol 3-phosphate
PLZF: promyelocytic leukemia zinc finger
RACK1: receptor for activated C kinase 1
SLAM: signaling lymphocyte-activation molecule
Tbkbp1: TBK-binding protein 1
TCR: T cell receptor
Th: T helper
Tsc1: tuberous sclerosis 1
Vps: vacuolar protein sorting.
